# Electrical Low-Frequency 1/*f*^γ^ Noise Due to Surface Diffusion of Scatterers on an Ultra-low-Noise Graphene Platform

**DOI:** 10.1021/acs.nanolett.1c02325

**Published:** 2021-09-07

**Authors:** Masahiro Kamada, Antti Laitinen, Weijun Zeng, Marco Will, Jayanta Sarkar, Kirsi Tappura, Heikki Seppä, Pertti Hakonen

**Affiliations:** † Low Temperature Laboratory, Department of Applied Physics, Aalto University School of Science, P.O. Box 15100, 00076 Aalto, Finland; ‡ Quantum systems, VTT Technical Research Centre of Finland Ltd., P.O. Box 1000, 02044 Espoo, Finland; ¶ QTF Centre of Excellence, Department of Applied Physics, Aalto University School of Science, P.O. Box 15100, 00076 Aalto, Finland; ▽ Microelectronics and quantum technology, VTT Technical Research Centre of Finland Ltd., QTF Centre of Excellence, 02044, Espoo, Finland

**Keywords:** 1/*f* noise, adsorption/desorption noise, graphene, neon, impurity clustering, diffusion

## Abstract

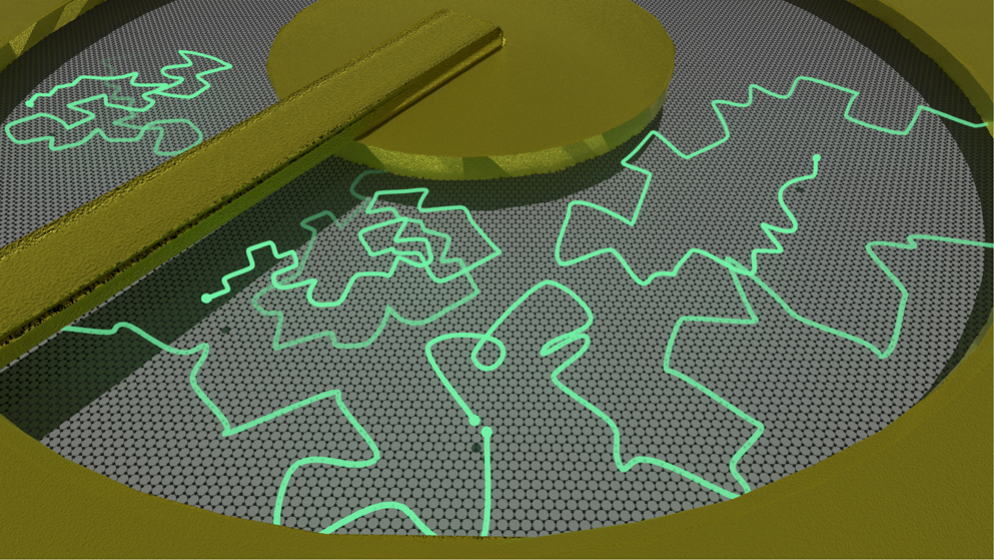

Low-frequency 1/*f* ^γ^ noise is ubiquitous, even in high-end electronic devices. Recently, it was found that adsorbed O_2_ molecules provide the dominant contribution to flux noise in superconducting quantum interference devices. To clarify the basic principles of such adsorbate noise, we have investigated low-frequency noise, while the mobility of surface adsorbates is varied by temperature. We measured low-frequency current noise in suspended monolayer graphene Corbino samples under the influence of adsorbed Ne atoms. Owing to the extremely small intrinsic noise of suspended graphene, we could resolve a combination of 1/*f* ^γ^ and Lorentzian noise induced by the presence of Ne. We find that the 1/*f* ^γ^ noise is caused by surface diffusion of Ne atoms and by temporary formation of few-Ne-atom clusters. Our results support the idea that clustering dynamics of defects is relevant for understanding of 1/*f* noise in metallic systems.

Quantum devices in nanotechnology are plagued by 1/*f* ^γ^ noise. Monolayer graphene devices are no exception, even though they have been found to exhibit ultralow noise.^1^ Suspended graphene has been found to provide the lowest noise, since it can be made nearly perfectly clean without the influence of defects in the nearby substrate, and it displays an ultrahigh mobility.^2^ Several physical mechanisms have been suggested as the origin of the 1/*f* ^γ^ noise in graphene, either via fluctuations of the chemical potential or directly via mobility fluctuations.^3−8^ In addition, contact noise has been found to be relevant in many cases,^2,9,10^ which may result from current crowding at the contacts.^11−13^

In general, the *ad hoc* models considered for graphene can be criticized, as they do not arise from a unified concept. The same issue, however, does pertain to various areas of 1/*f* noise.^14−17^ In this work, our goal is to test fundamental aspects of theories based on mobile impurities.^18−23^ We generate 1/*f* ^γ^ noise by adsorbing neon atoms onto a graphene membrane. We are employing suspended graphene as a platform for studying impurity-induced low-frequency noise, because the inherent 1/*f* ^γ^ noise level is exceedingly small in mechanically exfoliated, suspended graphene. Since the background impurity scattering is almost nonexistent for graphene electrons, even weak scatterers such as neon atoms may make a difference in the impurity scattering and thereby alter the noise substantially.

All solid materials contain structural defects that may diffuse around at room temperature. When temperature is lowered, the diffusion slows down, but it remains still visible all the way down to the quantum tunneling regime. We are interested in diffusion of defects or impurities and their possible role in scattering fluctuations due to clustering of defects/impurities. Diffusion influences the relative locations of impurities, which then affect the total scattering cross section (length in two dimensions) experienced by the charge carriers traversing the sample. Variation of the scattering will, in turn, lead to modification of resistance, and the fundamental question is whether this will lead to a 1/*f* ^γ^ noise spectrum with γ ≃ 1. The answer to this question bears significance also to quantum technology, as the interfacial states and adsorbate atoms present important noise sources for qubits.^24−26^

Owing to its large surface-to-volume ratio, graphene is always very susceptible to surface adsorbates, and even individual Hall resistance steps have been demonstrated due to single-atom adsorption events.^27^ It has experimentally been demonstrated that adsorbed gas molecules at room temperature will lead to Lorentzian noise spectra, with characteristics specific to the adsorbent species.^28^ Noble gases on graphite interact quite weakly with the substrate and the adsorbed atoms remain mobile at cryogenic temperatures. The most interesting regime is attained around 10 K where surface diffusion leads to fluctuations in the number and in the distribution of the atoms on the graphene membrane, while the desorption rate of Ne can be neglected. In this regime, we may test universal 1/*f* theories based on mobile impurities. Our results can also be employed to determine the adsorption energy of a Ne atom onto a graphene monolayer.

Figure 1 illustrates the basic influence of neon gas on our graphene samples fabricated using techniques described in refs (29) and (30). Figure 1a displays the measured low-frequency noise of a Corbino disk at 10 Hz as a function of temperature with and without neon. All the data reported in this work were obtained on a disk that had an inner and outer diameter of 1.8 and 4.5 μm, respectively (see the SI for details of the measurement setup). Without adsorbed Ne, the low-frequency noise increases approximately linearly with *T*, whereas the background level of *S*_*I*_/*I*^2^ with Ne is nearly constant up to *T* ≃ 20 K. At temperatures of 20–30 K, there is a strong peak in the noise, which can be attributed to the dynamics of neon atoms on the graphene surface, governed basically by adsorption/desorption processes; the *S*_*I*_/*I*^2^ reading at 10 Hz with Ne in Figure 1a corresponds to averaged noise obtained by fitting of a linear combination of 1/*f* ^γ^ and a Lorentzian spectrum to the data over 1–100 Hz. The smaller peak at *T* = 12–16 K may be due to residual hydrogen present in the system, but more likely, it is related to diffusion phenomena of neon atoms across the graphene sample. The quality of the sample is exemplified by the product *f* × *S*_*I*_/*I*^2^ ≃ 2 × 10^–10^, which is on par with the best noise levels reported so far.^2^

**Figure 1:**
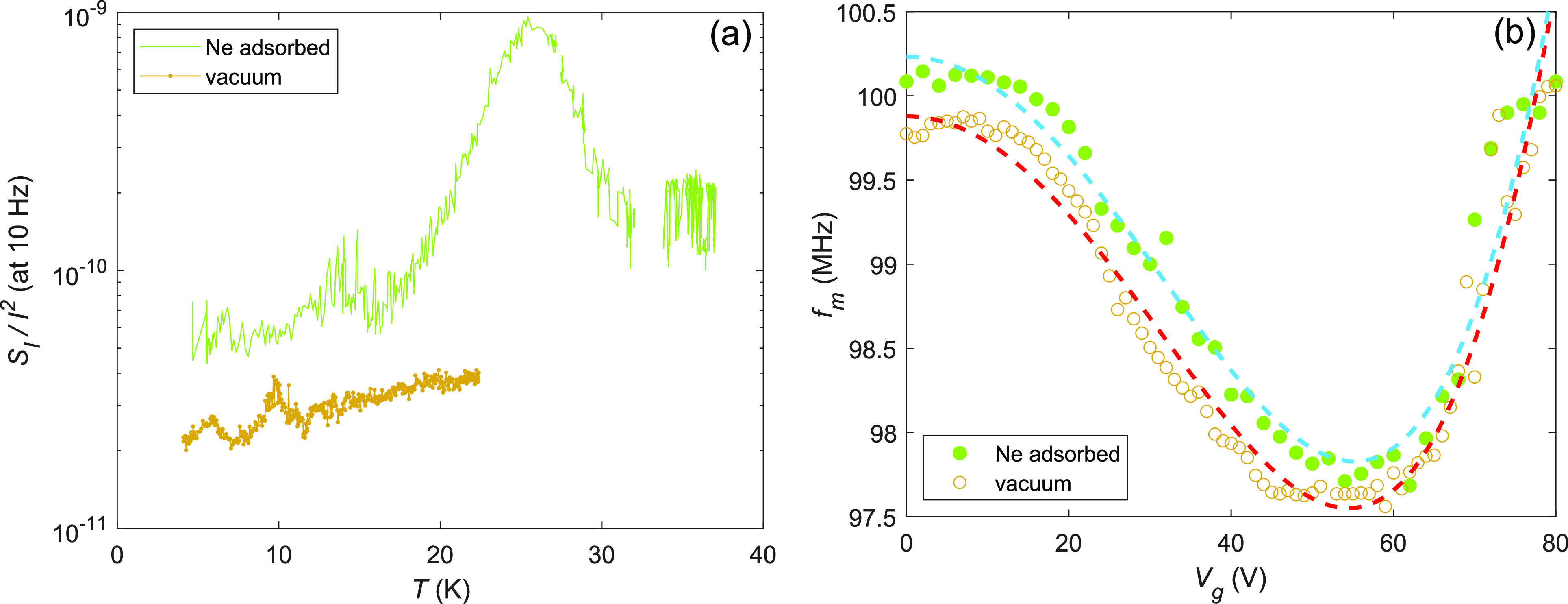
(a) Scaled current noise *S*_*I*_/*I*^2^ at 10 Hz as a function of *T* measured with (upper trace, green) and without (lower trace, orange) adsorbed Ne. (b) Mechanical resonance frequency *f*_*m*_ vs gate voltage *V*_*g*_ measured for Corbino disk with (green •) and without (○) adsorbed neon. The re-entrant-looking *f*_*m*_(*V*_*g*_) (half of W-shape) indicates a crossover from the initial-tension-dominated gate dependence to induced-tension-dominated behavior. The dashed curves are quartic fits to the data with (upper) and without neon (lower); the shift of the W-shape minimum to larger *V*_*g*_ is also a sign of enhanced tension by neon.

Figure 1b displays the mechanical resonance frequency measured for the graphene Corbino disk that was employed for the majority of our noise experiments. In fact, this device displayed several weak mechanical resonances with slightly different frequency, which we interpret as splitting of the fundamental mode into several local resonances due to nonuniform strain in the membrane. As seen in Figure 1b, the displayed resonant frequency is increased by 2‰ in the presence of adsorbed neon. This suggests that most of the Ne atoms will be adsorbed at the edges near the contact where the graphene–gold corner provides more advantageous adsorption conditions. The accumulation of neon to the edge can enhance the rigidity of the boundary condition, thereby increasing the frequency. However, it is also likely that the adsorbed neon will cause local strain, which enhances the frequency in spite of the increased mass. Similar behavior has been observed with ^3^He atoms on a carbon nanotube.^32^

The increase in strain is also corroborated in the shift of the minimum of the re-entrant-looking *f*_*m*_(*V*_*g*_) curve^33^ under the influence of neon. The re-entrant W-shape indicates a crossover from the built-in-tension-dominated behavior to gate-induced-tension dependence, the minimum frequency position of which moves to larger *V*_*g*_ with enhanced initial tension.

Strain variation on the atomic scale due to adsorbed atoms will lead to local pseudomagnetic fields as well as changes in the scalar potential, which can strongly modify the mobility^34,35^ and enhance the generation of 1/*f* ^γ^ noise.

Figure 2a displays low-frequency current noise measured on clean, suspended graphene. The data are displayed as *f* × *S*_*I*_/*I*^2^ so that pure *S*_*I*_/*I*^2^ ∝ 1/*f* form becomes a constant in this plot. A Lorentzian fluctuator spectrum ∝1/(*f*^2^ + *f*_*c*_^2^), on the other hand, would display a peak at the corner frequency *f* = *f*_*c*_. The data in Figure 2a display an almost constant value at each temperature, which means that the behavior is close to 1/*f* over the measured range, which covers frequencies from 1 to 100 Hz while temperature is varied across *T* = 4–23 K.

**Figure 2:**
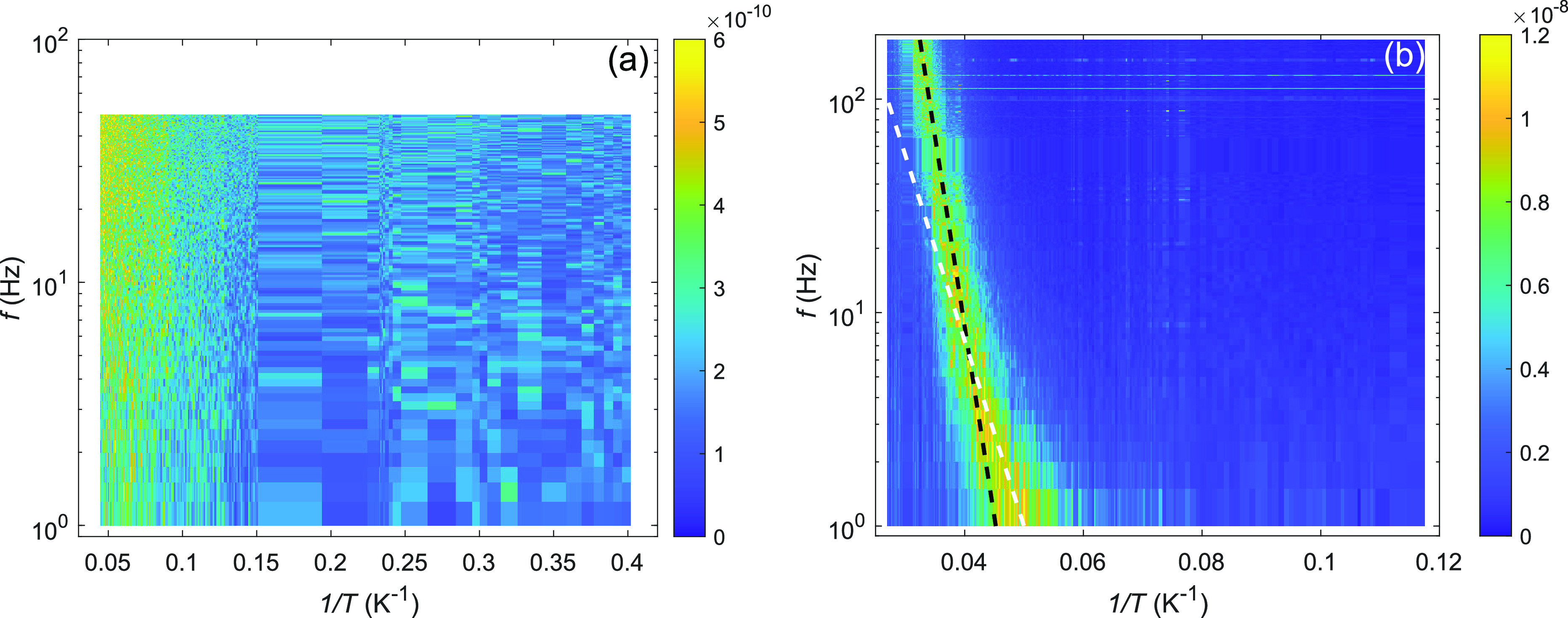
(a) Low-frequency noise in terms of dimensionless product *f* × *S*_*I*_/*I*^2^ for clean graphene on the inverse temperature 1/*T* vs frequency log *f* plane; the scale is ∼20× lower compared to measurements with neon adsorbates. (b) Scaled noise *f* × *S*_*I*_/*I*^2^ with neon atoms added to the sample depicted down to *T* = 8.5 K in order to enhance the visibility of features at high *T*. The dashed lines illustrate the cutoff frequency *f*_*c*_ (cf. eq 1) due to thermal activation, calculated for *f*_*c*_ = *f*_0_ exp(−*E*_*a*_/*k*_*B*_*T*) with the adsorption energy of *E*_*a*_/*k*_*B*_ = 410 K (the steeper one) and with the surface trap potential *E*_*b*_/*k*_*B*_ = 200 K (the low-temperature fit); Figure 3 of the SI displays a schematic of the surface potential with trapping states at the wall. The data were measured at the hole density of |*n*| ≈ 1.0 × 10^14^ m^–2^. For the amount of Ne, see the text.

The low-frequency noise is substantially stronger with adsorbed neon as seen in Figure 2b. The data plotted as *f*× *S*_*I*_/*I*^2^ display a maximum characteristic to a Lorentzian spectrum, in which the corner frequency *f*_*c*_ moves exponentially with inverse temperature. Exponential behavior exp[−*E*_*a*_/*k*_*B*_*T*] is expected for a thermally activated process following the Arrhenius law; here, *k*_*B*_ is the Boltzmann constant. Inspection of Figure 2b indicates two activation processes with slightly different activation energies. The fitted lines yield *E*_*a*1_/*k*_*B*_ = 410 K and *E*_*a*2_/*k*_*B*_ = 200 K. We identify *E*_*a*1_ = *E*_*a*_ as the adsorption energy of neon onto graphene, while the latter *E*_*a*2_ = *E*_*b*_ is identified as describing trap states at the boundary (see Sect III of SI). These trap states can act as expediters of trapping/detrapping behavior, potentially providing a similar resistance (current) noise mechanism as adsorption/desorption phenomena. Furthermore, the atomic graphene lattice potential has corrugations with saddle-point-like barriers separating nearby graphene hexagons. The ensuing diffusion barrier height for neon atoms amounts to approximately *E*_*d*_ = 32 K based on the values for graphite.^36,37^ Consequently, the observed current noise due to adsorbate dynamics on graphene is governed partly by surface diffusion with intermediate trapping and partly by adsorption/desorption, the relative weight of them depending on the temperature.^38,39^

Initially at low temperatures, we have thermally activated diffusion along the substrate, which becomes gradually influenced by desorption from the surface with increasing *T*. As discussed in the SI, we think that adsorption from the gas phase to the graphene membrane is limited due to lack of sticking sites except at the electrodes. The trap states at the boundary feed atoms back to the graphene surface at a rate governed by the relevant Arrhenius law, namely ∝exp[−*E*_*b*_/*k*_*B*_*T*], where *E*_*b*_ describes the depth of the trapping potential with respect to the potential surface in graphene (for a schematic picture, see the SI).

To describe the rate of change *Ṅ* in the number of the adsorbed Ne atoms on the graphene membrane, we employ a model with *N* mobile adsorbed atoms and a supply of *N*_*b*_ atoms captured by the additional trapping potential at the electrodes. Thus, our model has two coupled rate equations, one for *N* and one for *N*_*b*_ as outlined in the SI. The rates of exchange of atoms between the gas phase, the graphene membrane, and the surface trapping yield *N* and its fluctuation rate that governs the low-frequency scattering noise in the electronic transport. At high temperatures *T* > 25 K, desorption of atoms is faster than their diffusion, and direct adsorption/desorption processes govern the current noise. At low temperatures, on the other hand, exchange of atoms with the gas phase becomes irrelevant, and the dynamics of atoms is governed by release from the electrodes and ensuing diffusion on the Corbino disk.

Adsorption/desorption processes lead to Lorentzian noise spectrum given by
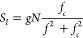
1where *g* reflects the strength of individual scatterers, *N* describes the number of particles involved in the process, and *f*_*c*_ is the frequency of desorption and adsorption processes which are equal at equilibrium. For thermal activation, we may write *f*_*c*_ = *f*_0_ exp(−*E*_*a*_/*k*_*B*_*T*) where *f*_0_ is the attempt frequency and *E*_*a*_ is the binding energy of Ne atoms on the graphene substrate. By fitting eq 1 to the data in Figure 2b, we obtain *E*_*a*_/*k*_*B*_ = 410 K with *f*_0_ in the range of 10^8^ s^–1^, which is rather low for an attempt frequency. However, low values for *f*_0_ have been obtained for surface diffusion of noble gases on metallic surfaces.^40^ Compared with neon adsorption energy on graphite *E*_*a*_/*k*_*B*_ = 350 K,^41^ our value is reasonable, taking into account the possible increase in interaction energy due to local deformation in a single-layer substrate. We emphasize that we always see a single Lorentzian line in the adsorption/desorption regime, never a collection of two level systems as seen for example in high-Ohmic graphene tunneling devices.^42^

With lowering temperature well below 25 K, the desorption rate of atoms becomes very small. Using the exponential activation fit to Figure 2b, the desorption rate  becomes ∼0.1 mHz at *T* = 15 K (with *f*_0_ = 10^8^ s^–1^). The diffusion time of Ne atoms across the Corbino ring varies between τ_*d*_ = 250···2 ms at temperatures *T* = 4···10 K (see Sect. III of the SI). Consequently, the probability of desorption from the surface during diffusion , and Ne atoms may diffuse across the Corbino disk without being desorbed during their flight time at *T* < 10 K. Therefore, diffusion of Ne atoms will govern fluctuations in the absorbent number and configuration patterns, which in turn, govern the low-frequency resistance noise in our system. The same diffusion processes on graphene govern also fluctuations in the number of atoms at the edge. This will lead to noise in the contact noise resistance *R*_*c*_, the separation of which is discussed in Sect. IV in the SI.

The diffusion process leads to a random walk type of noise where the characteristic frequency is given by the inverse of a typical random walk time,^43,44^ i.e., the inverse of diffusion time across the sample *f*_*c*_ = (2*πτ*_*d*_)^−1^ ,and *N* in eq 1 reflects now the average fluctuating number of atoms diffusing along the graphene sample. Hence, at *T* = 4–10 K, surface diffusion provides low-frequency noise in the range of investigated frequencies of 1–100 Hz. However, the diffusion of individual, noninteracting particles will lead to a noise spectrum of 1/*f*^1.5^ form.^44−47^ Only if there are additional correlations, for example, generated by clustering/declustering of neon atoms via thermally driven surface diffusion, the noise power spectral density may approach the 1/*f* spectrum. Basically, multipartite clustering dynamics leads to long-term memory effects, which modify the random walk nature of regular low-frequency diffusion noise.

In order to detail the noise in the desorption regime, Figure 3a, displays a few current noise spectra as *f* × *S*_*I*_/*I*^2^ is measured at different temperatures between 4–37 K. With increasing temperature, the peak in *f* × *S*_*I*_/*I*^2^ due to the cutoff frequency *f*_*c*_ shifts toward higher frequencies. The overlaid traces at temperatures *T* = 22.1 K, *T* = 25.0 K, and *T* = 29.8 K are fits calculated using eq 1 with *f*_*c*_ = 1.5, 12, and 120 Hz, respectively. These values hold remarkably well together with *f*_*c*_ = *f*_0_ exp(−*E*_*a*_/*k*_*B*_*T*) where *f*_0_ = 1.4 ± 0.25 × 10^8^ s^–1^. The absence of temperature dependence in the maximum amplitude of these three *f* × *S*_*I*_/*I*^2^ traces arises from the specific behavior of the sticking sites at the boundaries, i.e., due to the fact that the feed from the *N*_*a*_ trapped atoms is able to compensate the desorption rate of ∼*N* so that *N* = const. (see SI).

**Figure 3:**
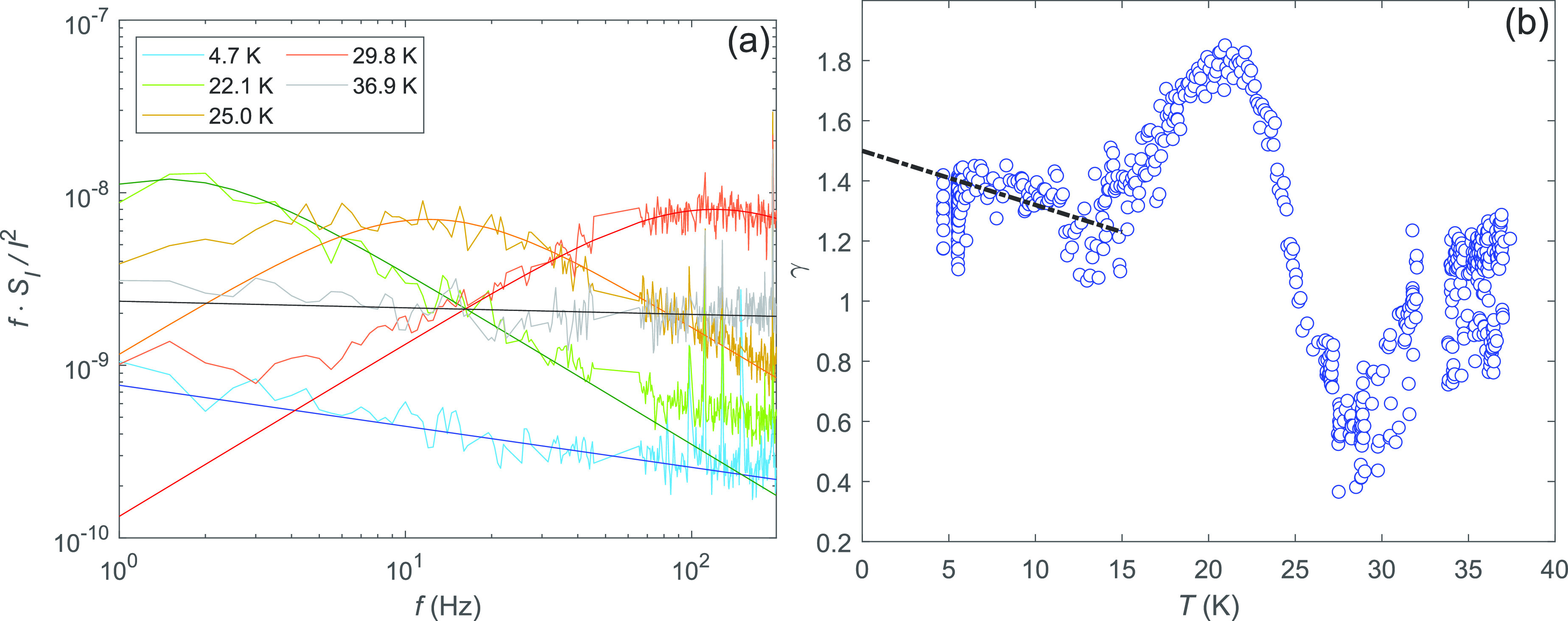
(a) Current noise spectral density multiplied by frequency *f* × *S*_*I*_/*I*^2^ measured at temperatures *T* = 4.7, 22.1, 25.0, 29.8, and 36.9 K. The three intermediate temperature traces are listed from bottom to top with regards to the high-frequency end (green, orange, red), with the overlaid traces obtained from eq 1 using *f*_*c*_ = 1.5, 12, and 120 Hz, respectively. The data at *T* = 4.7 K (blue) does not include a Lorentzian part, and it is described by a spectrum 1/*f* ^γ^ with γ = 1.24, while for the data at 36.9 K (gray/black), the exponent becomes γ = 1.04, indicating the smallness of fast-adsorption/desorption-process noise at low frequencies. (b) Exponent γ obtained from an overall *S*_*I*_ ∝ 1/*f* ^γ^ fit to the data in Figure 2b. The movement of *f*_*c*_ across the studied frequency range as a function of *T* is visible here as a change in γ from 1.8 to 0.5, equaling nearly the change from 2 to 0 expected from eq 1. The dashed line sketches the clustering-induced decrease of γ vs *T*.

Besides the Lorentzian-based fits of Figure 3a, we made power law fits to the noise using an arbitrary exponent: *S*_*I*_ ∝ 1/*f* ^γ^. Results of these unconditional fits to the data are illustrated in Figure 3b. The movement of *f*_*c*_ across the studied frequency range as a function of *T* is visible here as a nonmonotonic change in γ. A change in the exponent from 2 to 0 is expected on the basis of eq 1, but the data display a smaller swing: from 1.8 to 0.5. Adopting a commonly used criterion 0.5 < γ < 1.5 for 1/*f* noise, we may conclude that the presence of Ne is able to destroy the character of the noise when the sloped noise part ∝1/*f*^2^ is dominating. At *T* = 4–10 K, the noise spectrum is described by a single exponent γ = 1.2–1.4. One spectrum with γ = 1.24 measured at *T* = 4.7 K is displayed for reference in Figure 3a.

Noise induced by thermal diffusion has been demonstrated to lead to complex frequency dependence of noise.^48^ In our hopping transport, the complexity of neon diffusion depends on Ne–Ne interactions and the boundary conditions. If one calculates numerically the lifetime distribution of diffusing particles in a Corbino disk geometry with fully absorbing boundary conditions (see Sect. IV of SI), one obtains a noise spectrum of the form 1/*f*^1.5^, similar to the one-dimensional case.^44^ On the other hand, if we assume a probability of reflection of particles from the boundary (the relevant sticking site occupied), then we obtain γ > 1.5. Thus, without additional assumptions on time-dependent correlations among neon atoms, for example changes in scattering via clustering/declustering, the diffusion model is not sufficient for explaining the observed exponent γ = 1.2–1.4.

The central question of low-frequency noise due to diffusing adsorbates is the nature and strength of their mutual interactions. As discussed in Sect. I of the SI, it is known that Ne atoms have an attractive interaction on the order of ϵ/*k*_*B*_ ≃ 40 K which tends to stabilize 7^1/2^× 7^1/2^ commensurate structures on graphite. Thus, clusters of Ne atoms on graphene may form and they modify random diffusion by their interaction energy and by hopping restrictions imposed on the atoms sitting within the cluster. We have performed kinetic Monte Carlo (kMC) simulations (see Sect. V in SI) to investigate these effects and have found that the significance of clusters depends on temperature. At higher temperatures more atoms are diffusing and the interactions and clusters become more important. According to these kMC simulations, the resistance noise is first close to 1/*f* ^1.6^ at low *T*, at which mostly single atoms are diffusing, but there are also prominent fluctuations due to hopping in the boundary layer causing noise in the contact resistance *R*_*c*_ (for details, see SI Sects. V and VI). The noise becomes closer to 1/*f* ^1.2^ when temperature is increased by a factor of approximately two. These simulation results agree quite well with our data in Figure 3b, in which a decrease of γ from 1.4 to 1.2 is observed when temperature is varied between *T* = 4–10 K. This agreement strongly supports the importance of clustering of the adsorbates for generation of 1/*f* resistance noise, in similarity with adsorbate-induced flux noise in Al-based SQUIDs.

SQUID-based experiments indicate that fluctuations of unpaired microscopic surface spins with interactions are responsible for flux noise in superconducting quantum circuits.^49,50^ Interactions have also been found to lead to clustering of spins, which gives rise to interesting spin dynamics producing flux noise.^51,52^ Contrary to our resistance noise, the flux noise is argued to arise from spin flips (either single spin or a cluster) and spin diffusion due to interactions, not from lattice hopping of adsorbates as in our case. There is recent experimental evidence that surface spins due to adsorbed O_2_^24,25^ provide major contributions for flux noise in AlO_*x*_ tunnel junctions. Indeed, our results suggest that spatial clustering of such surface spins may play an important role also in flux noise.

In summary, we have investigated low-frequency noise in suspended graphene with and without adsorbed neon, in particular, in Corbino geometry, in which there are no free edges to interact with adsorbed gas atoms. We find ultralow 1/*f* noise amounting to *f* ·*S*_*I*_/*I*^2^ = (2 ± 0.5) × 10^–10^ for clean graphene at intermediate charge densities *n* = ± 7 ·10^11^ cm^–2^ at 4 K; the noise was enhanced by a factor of 3 when adding neon on the sample at these carrier densities.

At *T* ≫ 10 K, desorption of Ne atoms led to fluctuations in the number of mobile Ne atoms on the graphene surface, which caused a temperature-dependent characteristic fluctuation frequency *f*_*c*_ = *f*_0_ exp(−*E*_*a*_/*k*_*B*_*T*) that corresponds to adsorption energy *E*_*a*_/*k*_*B*_ = 410 K. At *T* < 20 K, the noise is governed by surface diffusion of Ne atoms, and models based on dynamical clustering of mobile impurities were tested at *T* = 4–10 K. Our work clearly demonstrates that a substantial amount of low-frequency noise is created by diffusing impurities on a 2D sample. The observed noise spectra around 4–10 K display a power law behavior 1/*f* ^γ^ with γ = 1.2–1.4. Fundamental agreement with our Monte Carlo simulations supports the conclusion that weakening of the frequency exponent from the single-particle diffusion noise with γ = 1.5 toward γ = 1 is due to the variation in the scattering cross section caused by clustering/declustering of the mobile neon impurities. Our results carry direct relevance for ultraclean graphene technologies,^53^ and they provide strong evidence that diffusing defects and their relative grouping/regrouping play a role in various systems displaying 1/*f* type of noise.

## Supporting Information Available

Video of Monte Carlo simulation at *k*_*B*_*T* = 1.2 (MP4)
Video of Monte Carlo simulation at *k*_*B*_*T* = 2 (MP4)
Additional information on experimental procedures, neon on graphite, thermal activation rate and potential barriers, random walk on Corbino disk geometry, Monte Carlo simulations and power spectral density of resistance fluctuations, and graphene noise vs contact noise (PDF)
