# Supplementary material for: Electrical Low-Frequency 1/fγ Noise Due to Surface Diffusion of Scatterers on an Ultra-low-Noise Graphene Platform
Source: Nano Lett. 2021 Sep 7;21(18):7637–43. doi: 10.1021/acs.nanolett.1c02325 (PMC8461652; doi:10.1021/acs.nanolett.1c02325)
Supplement: Supplementary file 3 — nl1c02325_si_003.pdf [file nl1c02325_si_003.pdf]

Supplementary information: Electrical low-frequency $1/f^{\gamma}$ noise due to surface diffusion of scatterers on an ultra low noise graphene platform

Masahiro Kamada,[†] Antti Laitinen,[†] Weijun Zeng,[†] Marco Will,[†] Jayanta Sarkar,[†] Kirsi Tappura,[‡] Heikki Seppä,[¶] and Pertti Hakonen[*,†,§]

†Low Temperature Laboratory, Department of Applied Physics, Aalto University School of Science, P.O. Box 15100, 00076 Aalto, Finland

‡ Microelectronics and quantum technology, VTT Technical Research Centre of Finland Ltd., QTF Centre of Excellence, 02044 Espoo, Finland

¶Quantum systems, VTT Technical Research Centre of Finland Ltd., P.O. Box 1000, 02044 VTT, Finland

§QTF Centre of Excellence, Department of Applied Physics, Aalto University School of Science, P.O. Box 15100, 00076 Aalto, Finland

E-mail: pertti.hakonen@aalto.fi

I Experimental procedures

In our experiments, we measured graphene Corbino disks, freely suspended by dissolving away a LOR sacrificial layer, onto which natural graphite had been exfoliated. Our sample fabrication techniques and illustrations of samples can be found in Refs. 1,2. Our most

extensive data set was taken using a Corbino disk with distance $L = 1.3$ μm between the electrodes (inner and outer diameters of 1.8 and 4.5 μm, respectively). The value of the gate capacitance $C_g = 1.5 \times 10^{-5}$ F/m^2 was determined using the spread of a Landau level fan diagram.[1] A close-up from a scanning electron microscope (SEM) image of a graphene Corbino sample is illustrated within the measurement schematics in Fig. S1.

Following the initial characterization at room temperature, the samples were mounted on a Bluefors LD250 dry dilution refrigerator and cooled down to $T = 10$ mK. Prior to low-frequency noise measurements, the graphene devices were current annealed at 4 K, at which cryopumping guaranteed a UHV level cryogenic vacuum. Cryopumping by the cryostat worked also at higher temperatures against impurity gases such as O_2 and N_2. In addition, current annealing was employed at regular intervals to clean neon away from the graphene surface, thereby verifying that the results indeed were caused by Ne atoms. The applied gate voltage V_g was employed to determine the charge carrier density according to $n = (V_g - V_g^D)C_g/e$, where V_g^D denotes the gate voltage value of the Dirac point. Measurements of zero-bias resistance $R_0(V_g)$ yielded the field-effect mobility $\mu_{FE} \sim 10^5$ cm^2/Vs using $\mu_{FE} = (\sigma - \sigma_0)/ne$, where the minimum conductivity σ_0 corresponds to the maximum of measured resistivity at the Dirac point. According to μ_{FE} determination, adsorption of Ne increased the mobility of graphene by ~ 30% at small charge densities $|n| \lesssim 2 \times 10^{14}$ m^{-2}. This indicates additional screening of Coulomb impurities by adsorbed Ne. The effect of Ne-induced charge screening/charge modulation could be removed by thermal annealing, which was repeatedly done during our experiments.

The measurement schematics is depicted in Fig. S1. The graphene disk with its gold electrodes, seen in the false color SEM image, is located on top of a 500-nm-thick lift off resist (LOR) layer. Strongly doped substrate Si^{++} acted as the gate for charge density control, coupled by capacitance C_g to the sample via a 500-nm vacuum gap and 280-nm layer of SiO_2. Bias-T components (a combination of inductance L and capacitance C in the schematics) were employed to separate the radio frequency (rf) section from the low frequency parts. The

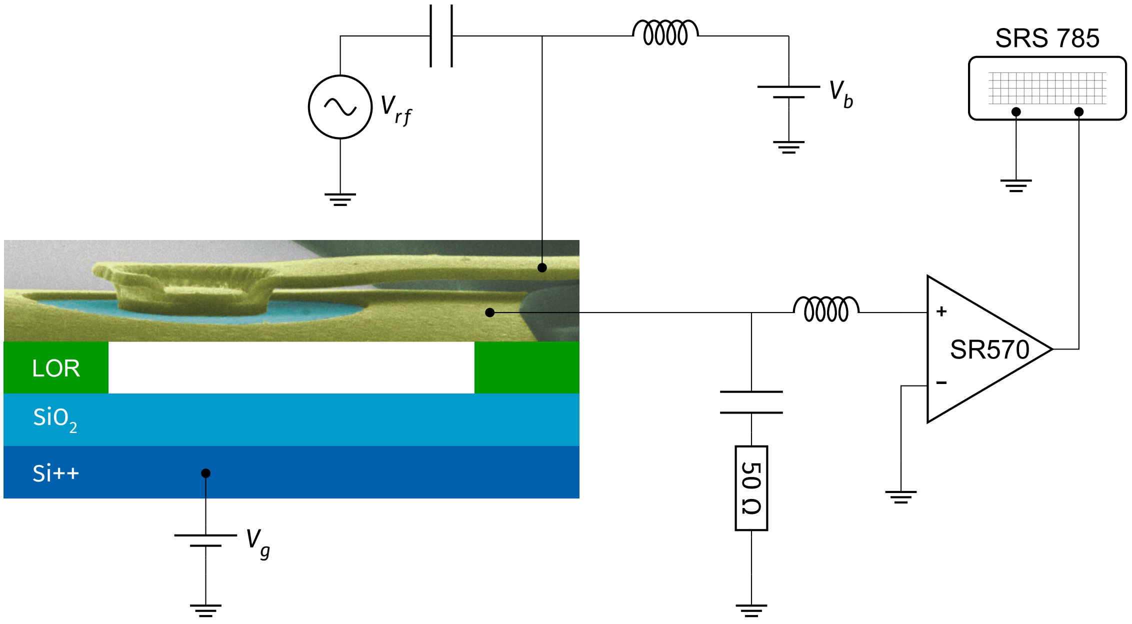

Figure S 1: Schematics of our measurement system. The graphene disk with its gold electrodes is depicted in a false color SEM image on top of the LOR layer. The outer diameter of the graphene Corbino disk is 4.5 μm. Bias-T components were employed to separate the radio frequency section used for mechanical vibrations from the low frequency parts employed to measure zero-bias resistance, IV characteristics, and noise. For details, see text.

rf-circuitry was needed to excite (using V_{rf}) and to record the mechanical resonance of the graphene membrane. Zero-bias resistance R_0 of the sample (consisting of a sum of graphene resistance R_{gr} and contact resistance R_c between graphene and gold) was measured at ~ 35 Hz using regular lock-in techniques keeping ac-voltage excitation on the order of temperature (i.e. ac voltage was about 0.4 mV at 4 K). The low frequency noise was measured under voltage bias up to V_b = 13 mV. A transimpedance amplifier (SR570, gain 10^5) was employed to track the current and the time trace of fluctuations was Fourier transformed using an SRS 785 FFT signal analyzer. The obtained $1/f^\gamma$ noise spectra, averaged for 180 s, consisted of 200 FFT points, spanning typically the frequency range of 1 – 200 Hz. The V_b^2 voltage dependence of the noise was verified over the whole employed frequency range. Dependence of the scaled low frequency noise power S_I/I^2 on various parameters was typically measured at 10 Hz. The intrinsic $1/f$ noise in our Corbino devices is quite low compared to all reported suspended graphene based devices of similar size, including our own earlier results

on suspended bilayer graphene.[3]

II. Neon on graphite

Atomic neon films on graphite provide a good starting point for understanding our sub-monolayer Ne films on graphene. An excellent overview of neon films on graphite is provided in Ref. 4. In general, quantum effects are a way smaller for neon atoms than for helium or hydrogen but they are still significant. For example, diffusion of Ne atoms is governed by quantum tunnelling at sub-Kelvin temperatures but, in our work at $T > 4$ K, thermally activated diffusion still dominates.

According to Ref. 5, Ne-Ne interaction has a binding energy of $\epsilon/k_B = 42$ K, while the binding energy to graphite $E_a/k_B = 380$ K, pretty close to the experimentally measured value of Ref. 6. Casimir-like adatom-adatom interaction via graphene electrons is also possible.[7] For commensurate solid structures, neon prefers a registered phase with $\sqrt{7} \times \sqrt{7}$ lattice and a four atom basis.[4,8] This means that interatomic interaction energy is minimized by local order, and that self-bound ordered regions, clusters, become possible, even though they would just provide a temporary structure. Parallel to this, the binding energy between neon atoms can be on the order of the diffusion barrier E_d, which means a significant energy scale considering the surface dynamics of atoms. These considerations provide justification for the parameter values employed in our kinetic Monte Carlo simulations targeting the clustering dynamics of adsorbed neon atoms (see Sect. IV below).

Our adsorbed neon films were prepared around 20 K with pressure $p \sim 10^{-4}$ mbar. On the basis of the empirical phase diagram for thin Ne films on graphite,[4,9] we conclude that no registered phases will be generated, and the behavior of the adsorbed atoms at $T = 10 - 37$ K will display gas or fluid like behavior. At temperatures $T < 10$ K, coexistence of solid phase and vapor becomes possible.[9] An immobile solid phase will be irrelevant for the generation of low frequency noise. Fluctuations of the shape of the solid, however, will contribute to the

noise in a similar fashion as fluctuations in the clustered atom regions seen in our simulations.

The addition of neon changed the dependence of graphene sample resistance, R, on the gate-induced carrier density n as illustrated in Fig. S2a. Clearly, $R(n)$ is more peaked near the Dirac point while the saturation conductance (inverse resistance) value becomes larger with Ne adsorption; this saturation value at $|V_g| \gg V_g^D$ is taken to correspond to the effective contact resistance R_c. Consequently, the data in Fig. S2a yields $R_c = 500\,\Omega$ and $405\,\Omega$ for clean and Ne-adsorbed states, respectively. With addition of Ne, the Dirac point of the sample also moved upwards nearly by 0.8 V in V_g, which has been taken into account when calculating the charge density.

Fig. S2b displays the field effect mobility calculated from $\mu_{FE}(n) = [\sigma(n) - \sigma_0(n_0)]/ne$ in which $\sigma(n)$ and $\sigma_0(n_0)$ denote the conductivity of the sample at carrier density n and at residual carrier density n_0 at the Dirac point. We display $\mu_{FE}(n)$ only for positive carriers because then R is not influenced by interfaces in the pnp doping structure at $V_g > V_g^D$. In the analysis, we determine separately the contact resistance for clean and Ne-adsorbed states and subtract R_c off from R before calculating the conductivity. Data in Fig. S2b indicate that addition of neon increases μ_{FE} by 30% at carrier densities $n = 1 \ldots 2 \times 10^{14}\,\mathrm{m}^{-2}$ which

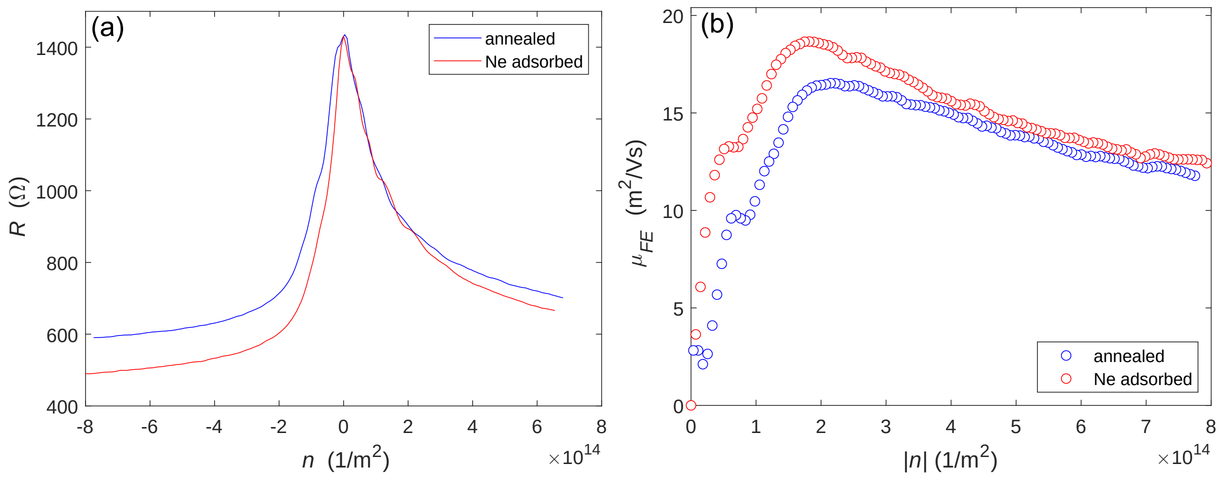

Figure S 2: a) Zero bias resistance R of the Corbino disk as a function of charge carrier density n measured in a state with adsorbed neon (red trace) and in a subsequent annealed, clean state (bluer trace). b) Field effect mobility μ_{FE} for holes obtained from the resistance data on the left at $n < 0$.

is the range where most of our noise data have been measured. This increase in mobility is quite close to the quoted 40% increase in μ_0 found from the magnetoresistance.

The asymptotic resistance value at large negative gate voltages in Fig. S2a is reduced by ~ 95 Ω by the addition of Ne. This indicates that resistance for hole transport near the gold contact is influenced by the adsorption of Ne and the hole contact resistance R_c^h (resistance not influenced by V_g) is lowered. Reduction in R_c^h can be assigned to Ne-induced strain that causes a change in the scalar and vector potentials in graphene in the neon-covered region. The strain-induced scalar potential modifies charge density near the contact and R_c^h becomes lowered as seen in Fig. S2a. For electronic transport at $V_g >> V_g^D$, the modification in the electronic contact resistance R_c^e is nearly zero.

We recently investigated magnetoresistance of the very same suspended Corbino disk.[10] Those experiments indicated that Coulomb scatterers at small carrier densities close to the Dirac point become better screened in the presence of a magnetic field, which led to a growing shift of the Dirac point with increasing magnetic field. Even stronger screening of charged impurities, together with a Dirac point shift, is observed in the presence of Ne adsorbates. In fact, the mobility μ_0 determined from magnetoresistance $\Delta R \propto (\mu_0 B)^2$ increased by 40% in the presence of Ne atoms, which is in line with the increase in μ_{FE} found from conductance. The strain induced by Ne atoms may increase the carrier density in graphene, which then improves screening of Coulomb impurities[11] in particular near the Dirac point and the mobility grows. Alternatively, strain-induced pseudomagnetic fields localize charge in such a way that residual impurities become screened. Thus, fluctuations in the strain-induced screening and pseudomagnetic fields are the central factors that make the influence of Ne so pronounced in the low-frequency noise in our experiments. Also contact regions are affected by similar effects as discussed in Sects. I and V in this SI.

Magnetoresistance investigations also allowed us to determine the contact resistance.[10] We employed the B^2 dependence of geometric magnetoresistance of graphene in Corbino geometry. The magnetoresistance yields mobility vs. gate voltage V_g that can be fitted

accurately using scattering by short ranged and Coulombic impurities. These scattering contributions together govern conductance $G(V_g)$ of bulk graphene. By subtracting the bulk contribution out, we obtain for the contact resistance $R_c \simeq 350 - 400\ \Omega$, nearly independent of the gate voltage. At large charge density the total resistance is dominated by R_c as is common in good quality samples. The actual value of R_c depended on the cleanliness of the sample and the presence of Ne on the sample. Note that the contact resistance is smaller with the presence of Ne on the sample.

III. Thermal activation and potentials

Our basic model for atomic Ne fluxes and trapping of atoms is illustrated in Fig. S3. The joint dynamics of gaseous neon and adsorbed atoms is governed by three different energy scales. The largest is the Ne-graphite adsorption energy E_a/k_B which is on the order 350 K.[6] For the atoms at the electrodes of the Corbino geometry there is an additional Ne-boundary adsorption energy E_b/k_B which amounts approximately to a few hundred Kelvin which is a typical noble gas-metal adsorption energy.[12] The smallest scale is related to the corrugation of the graphite potential E_d that provides the diffusion barrier of the neon atoms moving along graphene. We employ $E_d/k_B = 32$ K which has been reported for graphite.[13,14] In our simple modeling, we neglect the Ne-Ne interaction, but our Monte Carlo simulations do take this energy into account (see Sect. V).

When Ne atoms land on clean graphene, there are very few sticking sites for the atoms to get attached before they reach the boundary of the sample. Consequently, in our modeling we assume that neon atoms, becoming adsorbed from the gas phase, will become attached first to the wall potential marked by E_b in Fig. S3. We are interested in the time development of the number of mobile Ne atoms N on the graphene surface. Since neon atoms diffusing on graphene are supplied by N_b trapped atoms at the walls, we have two rate equations

governing the dynamics:

$$\dot{N}_b = -\frac{N_b}{\tau_s} + \dot{N}_a^{(gas)}, \tag{1}$$

$$\dot{N} = -\frac{N}{\tau_{ds}} - \frac{N}{\tau_d} + \frac{N_b}{\tau_s}, \tag{2}$$

where the upper equation describes the behavior at the wall and the latter one on graphene. N_b/τ_s denotes the rate at which atoms are released from the wall while $\dot{N}_a^{(gas)}$ gives the influx of atoms to the wall from the gas phase. The rate of change of Ne atoms on graphene $\dot{N}$ is given by desorption $-N/\tau_{ds}$, by diffusion and retrapping to walls $-N/\tau_d$, and by release from the walls at rate N_b/τ_s, where τ_i with $i = \{d, s, ds\}$ denotes the characteristic time scale for diffusion across the sample and retrapping to the walls, life time at the wall, and life time on graphene before desorption, respectively.

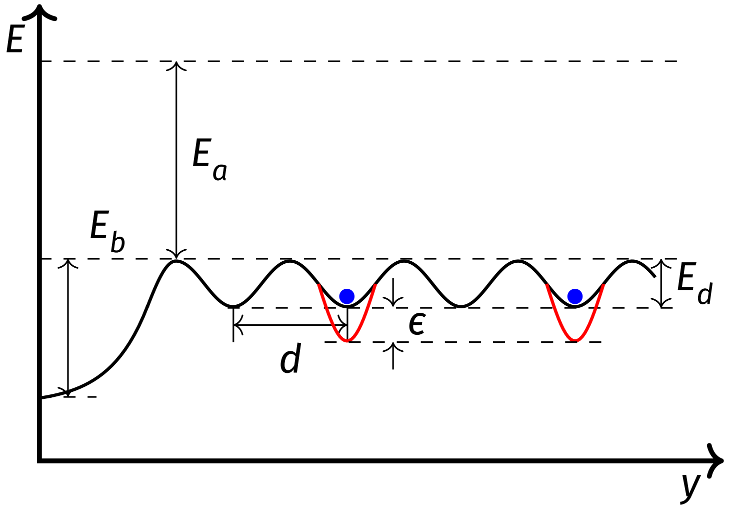

Figure S 3: Schematic potential for neon atoms on graphene near the gold electrode. Horizontal axis y denotes the distance from the gold wall. The energy scales, E_a, E_b, and E_d are explained in the text. The scale $d = 2.46$ Å marks the distance between the nearest hexagons of the graphene lattice. The two Ne atoms on the graphene lattice interact with energy ϵ which is the source of clustering, opposed by declustering due to thermal agitation.

In the steady state, $\frac{dN_b}{dt} = 0$ and $\frac{dN}{dt} = 0$, which yields

$$N_b = \dot{N}_a^{(gas)} \tau_s, \tag{3}$$

$$N = \frac{\tau_{ds}\tau_d}{\tau_{ds} + \tau_d} \frac{1}{\tau_s} N_b. \tag{4}$$

Using the definitions in Fig. S3, the time scales can be written as

$$\tau_s = \tau_s^{(0)} \exp\left(E_b/k_B T\right), \tag{5}$$

$$\tau_{ds} = \tau_{ds}^{(0)} \exp\left(E_a/k_B T\right), \tag{6}$$

$$\tau_d = \tau_d^{(0)} \exp\left(E_d/k_B T\right). \tag{7}$$

All time scales involve exponential activation type of behavior, but with quite distinct energy scales. The prefactors are related to inverse attempt frequencies f_0^{-1}; these frequencies are expected to be in the range of $f_0^{th} \simeq 10^8 - 10^{12}$ s^{-1}, but they vary depending on the actual curvature of the underlying potential and the temperature of the environment. However, since effective attempt frequencies have been observed to be even smaller than f_0^{th} with noble gases on metal surfaces,[12] we regard $\tau_s^{(0)}$ and $\tau_{ds}^{(0)}$ as fit parameters.

Diffusion of Ne atoms along the graphene is also thermally activated[12] and depends exponentially on temperature according to

$$D = D_0 \exp\left(-\frac{E_d}{k_B T}\right), \tag{8}$$

where D_0 denotes the prefactor which is expected to be on the order of $D_0 \sim \lambda^2 f_0^h$ in terms of the hopping length λ and the attempt frequency for hopping f_0^h. Using $f_0^h = 3.3 \cdot 10^{11}$ s^{-1} and $\lambda = 2.46$ Å, the hexagon-hexagon spacing, one obtains a value $D_0 \simeq 2.0 \times 10^{-8}$ m^2/s. Thus, we may estimate for the diffusion coefficient $D = 6.7 \times 10^{-12}$ m^2/s ($D = 8.1 \times 10^{-10}$ m^2/s) at 4 K (10 K) and the diffusion time becomes $\tau_d = \frac{L^2}{D} = \frac{L^2}{D_0} \exp\left(+\frac{E_d}{k_B T}\right) = 250$ ms (2 ms).

At high temperatures $T > 25$ K, desorption of atoms is faster than their diffusion across

the sample, and we have $\tau_{ds} \ll \tau_d$, while the opposite limit $\tau_{ds} \gg \tau_d$ is realized at low temperatures. In the former case, we have

$$N = \frac{\tau_{ds}\tau_d}{\tau_{ds} + \tau_d}\frac{1}{\tau_s}N_b \simeq \frac{\tau_{ds}}{\tau_s}N_b = \tau_{ds}\dot{N}_a^{(gas)} \simeq \text{const.}, \tag{9}$$

because τ_{ds} and $\dot{N}_a^{(gas)}$ have similar exponential temperature dependence. This is in accordance with the observed T-independence of the Lorentzian noise. Consequently, the sticking sites in our graphene device do not reside on the Corbino membrane, but at the boundary and they provide an effective, external ballast for the number of atoms of the graphene sheet as assumed in the analysis of the main text. If N would be governed by uniform graphene surface adsorption/desorption processes with a moderate sticking probability, a strong decrease of the maximum of $f \times S_I/I^2$ would be observed with lowering T.

In the low-temperature limit with $\tau_{ds} \gg \tau_d$, all the atoms from the gas phase have been adsorbed to surfaces, and we expect that the trapping states at the boundary are basically fully occupied, *i.e.* $N_b = N_S$, where N_S denotes the saturation amount at the wall.

$$N \simeq \frac{\tau_d}{\tau_s}N_S = \tau_d^{(0)}\exp\left(E_d/k_BT\right)\frac{N_S}{\tau_s^{(0)}}\exp\left(-E_b/k_BT\right) = \frac{\tau_d^{(0)}}{\tau_s^{(0)}}N_S\exp\left((E_d - E_b)/k_BT\right). \tag{10}$$

Since $E_d - E_b \sim -E_b$, there should be a strong T-dependence in N, and the number becomes reduced with lowering temperature. This will lead to more infrequent encounters between neon atoms and clustering of atoms becomes reduced at low T. This would then favor random walk type of noise which is detailed in Sect. IV next.

Finally, let us make a remark concerning the trapping potential E_b, which we have regarded as a constant. However, the potential at the boundary will have several energy levels for Ne atoms and the atoms on higher levels will experience a smaller trapping potential. Consequently, the effective trapping barrier E_b^{eff} for release of Ne atoms at low T may be smaller than $E_b = 200$ K, which would lead to a reduced decrease of N with lowering T than obtained from Eq. 10.

IV. Random walk on Corbino disk geometry

Random walk simulations were performed with a Corbino disk geometry of two concentric circles of 1.8 µm (inner) and 4.5 µm (outer) forming the contacts (see Fig. S4a). The particle starts at the outer contact and then moves on the graphene with a step size of 2.5 nm. Here a move is allowed with equal probability in the up, down, left or right direction. When the particle reaches the inner or outer contact it will get adsorbed. The amount of steps, which is proportional to the time diffusing on the graphene, is then recorded. Additionally, it is observed that 99.8% of the random walks that started at the outer contact also end there. In Fig. S4b 1000000 of such random walks are compared and a linear fit is applied to the first 1000 double logarithmic data points. The linear fit of $ax + b$ yields a slope of $k = -1.5$ which is in accordance with the one-dimensional calculation of Yakimov.[15] According to Ref. 15, the exponent for the spectrum is obtained from k as $\gamma = 3 + k = 1.5$.

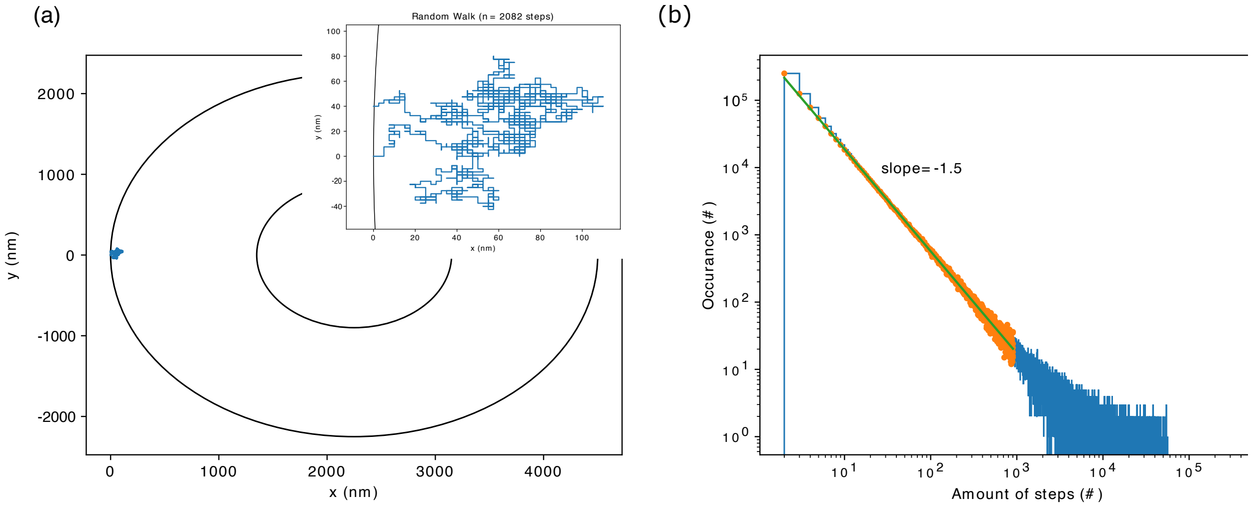

Figure S 4: a) Geometry of the Corbino disk simulation and example of a random walk. b) Distribution of 1000000 random walks (blue) on a 2.5 nm grid. Linear fit (green) to the first 1000 points (orange) yields a slope of −1.5.

Further, we investigated the possibility of reflection from the contacts back onto the graphene. With higher probability of reflection the fitted linear slope decreases to as low as 1.2 ($\gamma = 1.8$) at 75% chance of reflection.

V. Monte Carlo simulations and power spectral density of resistance fluctuations

Kinetic Monte Carlo simulations provide a powerful tool to investigate 2D dynamics in the presence of particle-particle interactions which are neglected in our basic diffusion calculations. In order to elucidate the role of Ne-Ne interactions as the origin of the $1/f^{\gamma}$ noise observed at $T = 4 - 10\,\mathrm{K}$, kinetic Monte Carlo (kMC) simulations were performed on a simplified model system imitating the Corbino geometry,[16].[17] The simulated system consists of a two-dimensional 50 by 50 square lattice with periodic boundary conditions applied to the left and right boundaries while rigid edges are assumed on the upper and lower boundaries corresponding to the electrodes of an infinitely large Corbino disk. We assume that all lattice sites are equivalent for impurity atoms, *i.e.* we neglect fully ripples on the graphene membrane, although such deformations might lead to clustering of impurities as such. We also neglect the Ne-Ne repulsion at short distances (see Sect. II) and allow the particles to occupy the nearest neighbour sites for computational simplicity.

In our starting configuration, 25 defects were placed on randomly selected lattice sites. Assuming vacancy diffusion type dynamics[16] - equivalent to the 2-state Ising model performing Kawasaki dynamics, where only one defect can occupy one lattice site at a time and the defects are allowed to move via thermally activated diffusional hops to any of the eight nearest non-defect lattice sites with the rates governed by the following equations:

$$r = f_0 \exp\left(\frac{-E_d}{k_B T}\right), \text{ if } \Delta E \leq 0, \tag{11a}$$

$$r = f_0 \exp\left(-\frac{E_d + \Delta E}{k_B T}\right), \text{ if } \Delta E > 0, \tag{11b}$$

where r is the average rate of a hop to one of the neighboring sites, f_0 is the attempt frequency, ΔE is the change in the system energy and E_d is the activation energy for the diffusional hop, and T is the temperature. ΔE is determined by the coordination number

between the neighboring defects assuming that when the coordination number increases by one, $\Delta E = -2$, and when it decreases by one, $\Delta E = 2$, and so on. Thus, according to Eqs. 11 the rate of cluster formation is somewhat higher than that of the dissociation and the rates depend on temperature. In our present simulations, simple energy relations were applied also for the other parameters: $f_0 = 1$, $E_d = 4$ and $k_B T = 1.2$ or $k_B T = 2$.

After the kMC simulations, the produced time series of the positions of the moving defects were used as an input to calculate the corresponding time series of the fluctuating resistance of the system. A minimal model for impurity scattering was employed to estimate the induced resistance change due to the diffusing particles: The resistance of a defect site was taken to be much lower, or alternatively much larger,[1] than that of the background lattice.[18] In our finite element method (FEM) calculations,[19] we assigned a 10^5 times smaller (or larger) value for the conductivity at each defect site. Most of our kMC simulations were performed using increased resistance at the defect sites, because atom clusters will act as real scattering centers which eventually win over the screening effects of single atoms. The resistance at every time step was determined by applying a small DC current from the lower electrode to the upper one and measuring the corresponding voltage. Finally, the power spectral density of the resistance fluctuations (PSD) was calculated and compared to the experimental results.

Fig. S5 shows the power spectral density of resistance fluctuations for two simulations performed at different temperatures, $k_B T = 1.2$ and $k_B T = 2$, with strong resistance at the scattering sites; practically the same behavior is obtained by setting large conductance at the impurity sites. In the case of $k_B T = 1.2$, the resistance fluctuations follow the power law of $1/f^\gamma$ with $\gamma \sim 1.6$ near the inspected frequency range, while for the higher temperature the power of $\gamma \sim 1.2$ is exhibited. Based on the kMC simulations, the dynamics of the defects as a function of time was further studied by extracting the average number of defects not in contact with the electrodes showing the average of 3.4 and 13 for $k_B T = 1.2$ and $k_B T = 2$,

[1] In the experiments, the presence of adsorbed Ne atoms leads to reduced scattering owing to improved screening of Coulomb impurities.

respectively. This implies that the average time the defects spend at the rigid edges, imitating the contact electrodes of the Corbino disk, is significantly longer at the lower temperature, reducing the effective number of mobile defects on graphene. This can also be seen in the attached videos and the representative snapshots of the trajectories shown in Fig. S6.

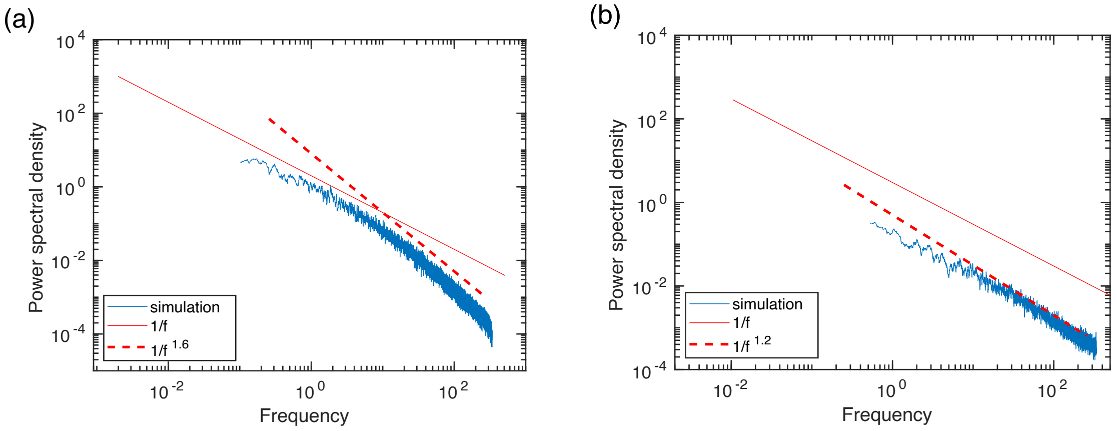

Figure S 5: Power spectral density of resistance fluctuations for two different temperatures: (a) $k_BT = 1.2$ and (b) $k_BT = 2$. The frequency scale depends on the attempt frequency, here scaled to correspond to $f_0 \sim 10^8$ s^{-1} on the Corbino disk.

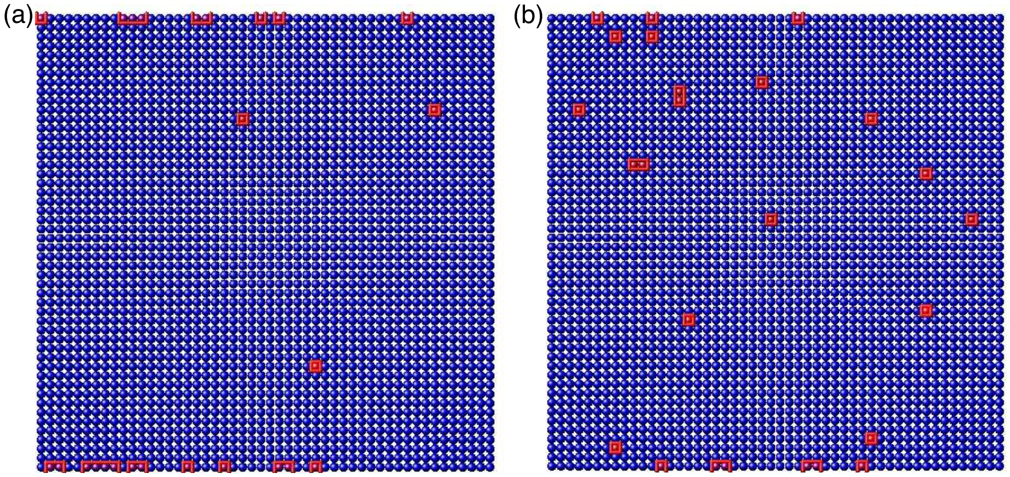

Figure S 6: Snapshots of a trajectory of the defects on the lattice: (a) $k_BT = 1.2$ and (b) $k_BT = 2$ (for videos see supplementary material mc140_movie_small_dt.mp4 ($k_BT = 1.2$) and mc147_movie_small_dt.mp4 ($k_BT = 2$))

The comparison of Figs. S5 and S6 supports the conjecture that the absorbing boundaries of the electrodes have a significant influence on the observed noise spectra. In more detail, the simulations indicate the importance of cluster formation and dissociation especially to

the observed frequency specific power, γ, of $1/f^\gamma$: the average number of mobile clusters is more than four times higher for $k_BT = 2$, corresponding to $\gamma \sim 1.2$, as compared to the case of $k_BT = 1.2$, where single mobile defects dominate the dynamics at the inspected high frequency range, corresponding to $\gamma \sim 1.6$. At lower frequencies where longer correlations become visible, the PSD curves approach the form of $1/f$ noise. In the case of $k_BT = 2$ (Figs. S5(b) and S6(b)), the clustering based correlations are visible also at higher frequencies due to the larger number of mobile clusters.

VI. Graphene noise vs. contact noise

In the main paper, we considered the $1/f^\gamma$ noise as coming from graphene without trying to separate the exact origin of the noise, whether it comes purely from graphene or whether it is also related to electrical contacts. As is well known, the resistance in high quality graphene samples originates mostly from the contacts, and the same could happen with the $1/f^\gamma$ noise. The separation of noise contribution from contacts has been discussed in clean graphene in Ref. 20 in presence of incoherent noise sources. Specific features of the measured $S_I(V_g)$ could be related to contact noise S_I^c and to the graphene noise S_I^{gr}. In particular, a M-shaped $S_I(V_g)$ curve with leveling off at large charge densities could be explained using the incoherent noise source model, in which the dip in the noise is related to S_I^c and to the coexistence of electrons and holes.[20] Moreover, the contact noise governs the leveling off of $S_I(V_g)$ at large carrier densities.

Fig. S7 displays current noise $S_I(V_g)$ data measured with adsorbed Ne atoms at $T = 4\,\mathrm{K}$ and $T = 20\,\mathrm{K}$. The data display a well-defined minimum of noise at the Dirac point, followed by a maximum in $S_I(V_g)$ at gate voltage $V_g \simeq \pm 10\,\mathrm{V}$, and finally a clear decrease of noise at $|V_g| > 10\,\mathrm{V}$ which tends to saturation when $|V_g| \to 50\,\mathrm{V}$. The behavior thus follows exactly the V_g dependence outlined in the analysis of Ref. 20. Consequently, we can conclude that the division between contact noise S_I^c and graphene noise S_I^{gr} is qualitatively similar in the

presence of adsorbed Ne as in clean graphene. From the data at $T = 4\,\mathrm{K}$, however, we may infer that the contact noise becomes asymmetric with respect to electrons and holes: electron conduction is seen to have smaller noise than the hole conduction at $V_g < 0$. Comparing with Fig. S2, we see that the change in contact resistance due to Ne atoms is stronger in the hole carrier regime, *i.e.* under similar conditions as S_I^c. Hence, we conclude that both contact noise and graphene noise are influenced by adsorbed Ne. Apart from local pseudomagnetic fields due to individual adsorbed atoms, the noise in both cases is due to changes in the local doping of graphene due to variation in Ne-induced scalar potential. Our kMC analysis with Corbino like boundary conditions includes significant clustering of atoms at the contacts and thereby noise due to changes in the boundary layer are an integral part of our numerical noise analysis. On broader scale, the origin of the contact noise is the same as that of graphene, even though the presence of the Au side wall changes the basic conditions for clustering.

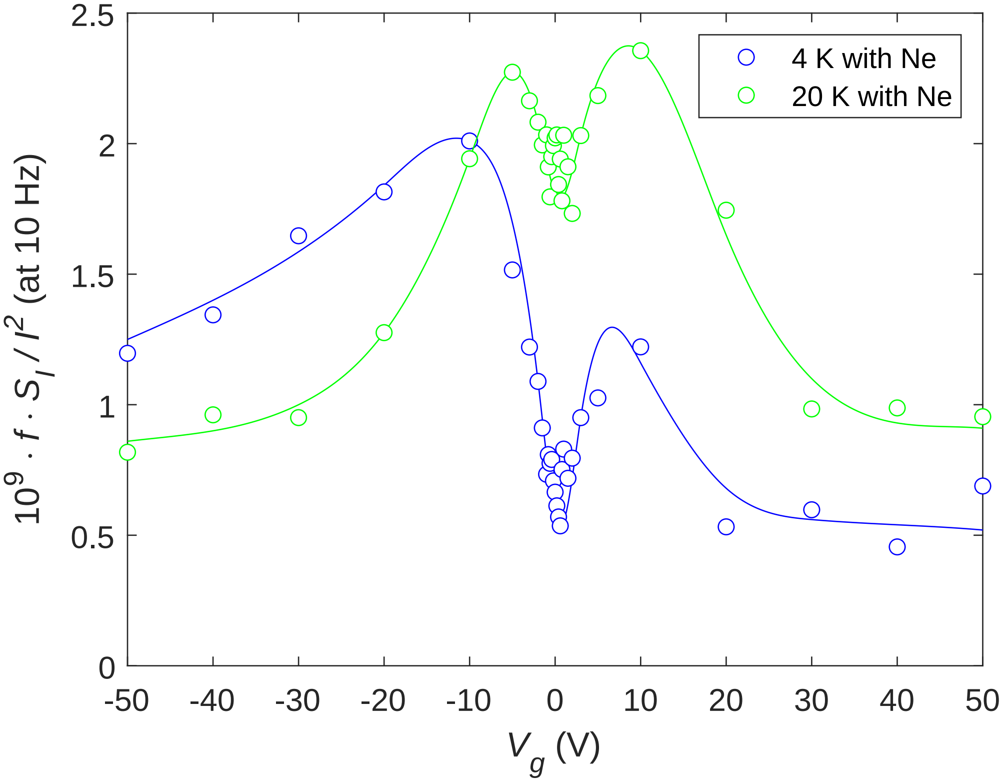

Figure S 7: Gate voltage dependence of scaled current noise $f \cdot S_I/I^2$ at 10 Hz measured with adsorbed Ne atoms at $T = 4\,\mathrm{K}$ (blue) and $T = 20\,\mathrm{K}$ (green) . The overlaid curves are to guide the eyes.

References

(1) Kumar, M.; Laitinen, A.; Hakonen, P. Unconventional fractional quantum Hall states and Wigner crystallization in suspended Corbino graphene. *Nature Communications* **2018**, *9*, 2776.

(2) Laitinen, A.; Paraoanu, G. S.; Oksanen, M.; Craciun, M. F.; Russo, S.; Sonin, E.; Hakonen, P. Contact doping, Klein tunneling, and asymmetry of shot noise in suspended graphene. *Physical Review B* **2016**, *93*, 1–14.

(3) Kumar, M.; Laitinen, A.; Cox, D.; Hakonen, P. J. Ultra low $1/f$ noise in suspended bilayer graphene. *Applied Physics Letters* **2015**, *106*, 263505.

(4) Bruch, L. W. *Physical adsorption: forces and phenomena*; Dover Publications: Mineola, N.Y, 2007.

(5) Gatica, S. M.; Cole, M. W. To wet or not to wet: that is the question. *Journal of Low Temperature Physics* **2009**, *157*, 111–136.

(6) Antoniou, A. A. The adsorption of neon on graphitized carbon in the submonolayer and multilayer region between 1.5 and 30°K. *The Journal of Chemical Physics* **1976**, *64*, 4901–4911.

(7) Shytov, A. V.; Abanin, D. A.; Levitov, L. S. Long-range interaction between adatoms in graphene. *Physical Review Letters* **2009**, *103*, 016806.

(8) Huff, G. B.; Dash, J. G. Phases of neon monolayers adsorbed on basal plane graphite. *Journal of Low Temperature Physics* **1976**, *24*, 155–174.

(9) Calisti, S.; Suzanne, J.; Venables, J. A LEED study of adsorbed neon on graphite. *Surface Science* **1982**, *115*, 455–468.

(10) Kamada, M.; Gall, V.; Sarkar, J.; Kumar, M.; Laitinen, A.; Gornyi, I.; Hakonen, P. Strong magnetoresistance in a graphene Corbino disk at low magnetic fields. *Physical Review B* **2021**, accepted.

(11) Hwang, E. H.; Adam, S.; Das Sarma, S. Transport in chemically doped graphene in the presence of adsorbed molecules. *Phys. Rev. B* **2007**, *76*, 195421.

(12) Barth, J. Transport of adsorbates at metal surfaces: from thermal migration to hot precursors. *Surface Science Reports* **2000**, *40*, 75 – 149.

(13) Carlos, W. E.; Cole, M. W. Interaction between a He atom and a graphite surface. *Surface Science* **1980**, *91*, 339–357.

(14) Cole, M. W.; Frankl, D. R.; Goodstein, D. L. Probing the helium-graphite interaction. *Rev. Mod. Phys.* **1981**, *53*, 199–210.

(15) Yakimov, A. V. Impurity and defect diffusion and flicker fluctuations in number of carriers in conductive media. *Radiophysics and Quantum Electronics* **1980**, *23*, 170–174.

(16) Plimpton, S. J.; Battaile, C. C.; Chandross, M. E.; Holm, E. A.; Thompson, A. P.; Tikare, V.; Wagner, G. J.; Webb III, E. B.; Zhou, X. W.; Garcia Cardona, C.; Slepoy, A. Crossing the mesoscale no-mans land via parallel kinetic Monte Carlo. **2009-6226**,

(17) Plimpton, S.; Thompson, A.; Slepoy, A. https://spparks.sandia.gov/. 2021.

(18) Lee, H.; Cho, D.; Shekhar, S.; Kim, J.; Park, J.; Hong, B. H.; Hong, S. Nanoscale direct mapping of noise source activities on graphene domains. *ACS Nano* **2016**, *10*, 10135–10142.

(19) COMSOL Multiphysics®. 2021; `https://www.comsol.com/`.

(20) Kamada, M.; et al., $1/f$ noise due to mobility fluctuations in graphene Corbino disk at low magnetic fields. *to be submitted*
